# Report on the First African Swine Fever Case in Greece

**DOI:** 10.3390/vetsci8080163

**Published:** 2021-08-11

**Authors:** Georgia D. Brellou, Panagiotis D. Tassis, Emmanouela P. Apostolopoulou, Paschalis D. Fortomaris, Leonidas S. Leontides, Georgios A. Papadopoulos, Eleni D. Tzika

**Affiliations:** 1 Department of Pathology, Faculty of Health Sciences, School of Veterinary Medicine, Aristotle University of Thessaloniki, 54627 Thessaloniki, Greece; emmaapos@vet.auth.gr; 2 Farm Animals Clinic, Faculty of Health Sciences, School of Veterinary Medicine, Aristotle University of Thessaloniki, 54627 Thessaloniki, Greece; ptassis@vet.auth.gr (P.D.T.); eltzika@vet.auth.gr (E.D.T.); 3 Laboratory of Animal Husbandry, Faculty of Health Sciences, School of Veterinary Medicine, Aristotle University of Thessaloniki, 54124 Thessaloniki, Greece; fortomap@vet.auth.gr (P.D.F.); gpapadop@vet.auth.gr (G.A.P.); 4 Laboratory of Epidemiology, Biostatistics and Animal Health Economics, School of Veterinary Science, University of Thessaly, 43132 Karditsa, Greece; leoleont@vet.uth.gr

**Keywords:** African swine fever virus, domestic swine, pathology, septicemic disease, outbreak, Greece

## Abstract

African swine fever (ASF) poses a major threat to swine health and welfare worldwide. After several European countries have reported cases of ASF, Greece confirmed officially the first positive case on 5 February 2020. The owner of a backyard farm in Nikoklia, a village in Serres regional unit, Central Macedonia, reported a loss of appetite, weakness, dyspnea, and the sudden death of 6 domestic pigs. Necropsy was performed in one gilt and findings were compatible with acute to subacute septicemic disease. Predominantly, hyperemic enlargement of spleen and lymph node enlargement and/or hemorrhage were observed. Description of vague clinical signs by the farmer suggested a limited resemblance to ASF-acute infection. However, the disease could not be ruled out once septicemic condition including splenomegaly, was diagnosed macroscopically at necropsy. In addition, considering the farm’s location near to ASF protection zones, a further diagnostic investigation followed. Confirmation of the disease was obtained using a series of diagnostic tests on several tissue samples. Further clinical, molecular, and epidemiologic evaluation of the farm was performed. According to the contingency plan, authorities euthanized all 31 pigs on the farm, whilst blood testing revealed ASF virus infection. Further emergency measures were implemented to contain the spread of the disease.

## 1. Introduction

African swine fever virus (ASFV) is a large, double-stranded DNA virus in the Asfarviridae family, genus Asfivirus. ASFV is regarded as the only DNA virus that can be classified as an ARBO (arthropod borne) virus [1]. It infects only members of the Suidae family of all age groups and is a harmless companion of the warthog (Phacochoerus aethiopicus) and the bushpig (Potamochoerus porcus) [2,3]. The virus can be transmitted via direct contact with infected animals or indirectly through consumption of infected pork products, through infected soft ticks (*Ornithodoros* spp.) bites, or after contact with fomites contaminated with virus-containing materials/biological fluids such as blood, feces, urine, or saliva [4]. Carcasses of infected wild boars maintain the live virus for a long time, especially during winter, allowing for indirect transmission when in contact with susceptible wild boars [5].

African swine fever (ASF) is a highly contagious viral hemorrhagic disease of domestic pigs and the wild boar population with a severe economic impact on pork production and meat supply [6,7]. It is an acute-to-chronic, febrile disease characterized by high fever, cutaneous hyperemia, abortions, edema, and hemorrhage in internal organs, particularly lymph nodes [8]. Up today, due to the lack of treatment or vaccine development, the early detection and implementation of strict preventive measures constitute the only way to eliminate the disease [9]. Genetic diversity and the very large and complex genome of the ASF virus increase the difficulty of vaccine development, however, efforts with live attenuated or subunit vaccines from various research groups, have started with respective pilot studies [10,11,12,13].

Clinical and gross features of ASF depend on the virulence of the virus isolate, the route and dose of infection, and the host’s immunological status [14]. However, a recent experimental study in Poland by Walczak et al. 2020, suggested that the same virus isolate might cause various clinical forms of the disease [15]. ASFV strains are usually classified as highly, moderately, and low virulent [16]. Four clinical forms of ASF have been described up to today. Peracute ASF usually occurs as a result of highly virulent strains. In that form, pigs die suddenly without any sign of the disease, or infected pigs develop loss of appetite, lethargy, high fever and die 1–4 days post-infection [14,17]. Moderately or highly virulent isolates are accountable for the development of the most usual form of the disease, the acute ASF, which includes vomiting, mucoid to bloody nasal discharges, melena, inactivity, and a tendency to crowding, as well as erythema or cyanosis of the skin, and abortion in sows [14,18,19,20]. Affected farms may show up to 100% mortality rate seven days after the initial clinical signs develop. The subacute form of ASF occurs after the infection by moderately virulent strains and clinical features resemble those of acute ASF, but they are less severe [14]. Infection by moderate-to-low virulence strains leads to the chronic form of ASF [19,21]. However, this form has not been detected in countries where moderately and highly pathogenic ASFV strains have been present for a long time. It has probably been associated with low virulent strains of ASF employed in early vaccine trials carried out in the Iberian Peninsula in the 1960s [14].

At the post-mortem examination, the most remarkable finding of acute ASF is the hyperemic splenomegaly followed by multifocal hemorrhagic lymphadenitis observed mainly in renal and gastrohepatic lymph nodes [17,19]. Severe pulmonary edema is a characteristic finding which occurs in animals affected by highly pathogenic strains [22,23,24]. Additionally, petechial hemorrhages may be detected in the cortex and renal pelvis of the kidneys as well as in the epicardium, the endocardium, the pleura, and the mucosa of the urinary bladder [19,25,26,27,28,29]. In the subacute form, hemorrhage and edema are more intense [20,25]. Features of chronic ASF are mainly associated with secondary bacterial infections. These include fibrinous pleuritis and/or pericarditis, pleural adhesions, necrotic or chronic pneumonia, fibrinous arthritis, and necrosis of the skin, tongue, and tonsils [18,30].

In Europe, the first outbreak of the disease occurred in the second half of the 20th century. In the first half of the 20th century, ASF cases were primarily restricted in Africa. The infection was eradicated via drastic control measures from all non-African countries, except the Italian island of Sardinia. In 2007, the ASF virus was introduced into the Caucasus and Eastern Europe where it has become endemic [31,32]. Since 2018, several Asian countries have also reported ASF virus infections [33]. From June 2019 to January 2020, the neighboring country Bulgaria reported 225 and 49 ASF cases in wild and domestic pigs, respectively. Some of those cases were detected close to the Greek borders [34]. Therefore, Greek authorities have established protection and supervision zones in two regional units next to the borders (Xanthi and Drama prefectures) since November 2019. The objective of this article is to describe the clinical, pathological, and epidemiological features of the first African swine fever case on a backyard farm in Greece, in 2020.

## 2. Case Presentation

### 2.1. Postmortem Examination

On 3 February 2020, the carcass of an 8-month-old gilt was admitted to the Laboratory of Pathology, School Veterinary Medicine, Faculty of Health Sciences, Aristotle University of Thessaloniki. The owner of the farm reported that the animal died after 6 days of anorexia, weakness, and dyspnea and claimed that 6 domestic pigs had died the past two weeks and 3 more were sick with similar clinical signs. The pigs resided in an olive grove in two adjacent, but separate fenced holdings. Cases expanded gradually from the first to the second holding. According to the farmer, all pigs were not vaccinated, and they had received antibiotics as treatment for the above-mentioned clinical symptoms, which were attributed to a possible “respiratory disease”. Like for any animal presented for necropsy, biosecurity measures were implemented to prevent the spread of potential pathogens and a post-mortem examination was carried out.

**Figure 1:**
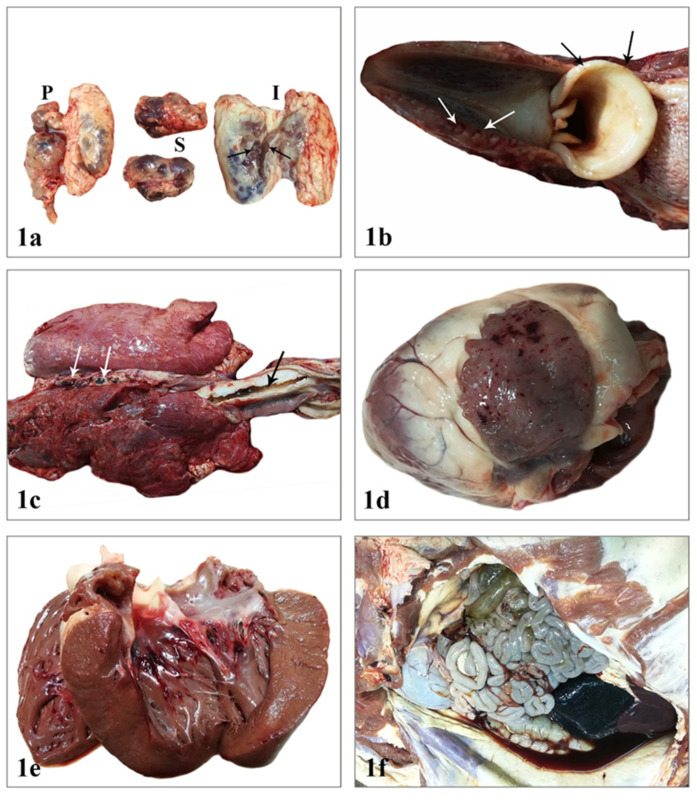
(**a**). Enlarged superficial cervical (prescapular) (P), submandibular (S), and inguinal (I) lymph nodes. The submandibular lymph nodes appear also diffusely hyperemic and the inguinal on the cut surface are characterized by hemorrhage at the periphery of lymphoid follicles, probably in the marginal zone (arrows). (**b**). A few petechiae are observed on the epiglottis (black arrows), the tonsils are mildly enlarged and on the cut surface are shown formations similar to those described in Figure 1a for inguinal lymph nodes (white arrows). (**c**). The right lung architecture is locally distorted because of fibrosis and the presence of symphysis between visceral and parietal pleura. Lungs are diffusely hyperemic and frothy material (pulmonary edema) occupies the tracheal lumen (black arrow). The caudal mediastinal lymph nodes are hemorrhagic (white arrows). (**d**). Multiple subepicardial petechial and ecchymotic hemorrhages are noted in the right auricle of the heart. (**e**). Hemorrhages are located mostly in the subendocardium of the left ventricle. (**f**). The peritoneal cavity bears serosanguineous content with a few filaments of fibrin. The spleen is discernible on the right side of the abdomen due to enlargement and presents dark red to black color.

**Figure 2:**
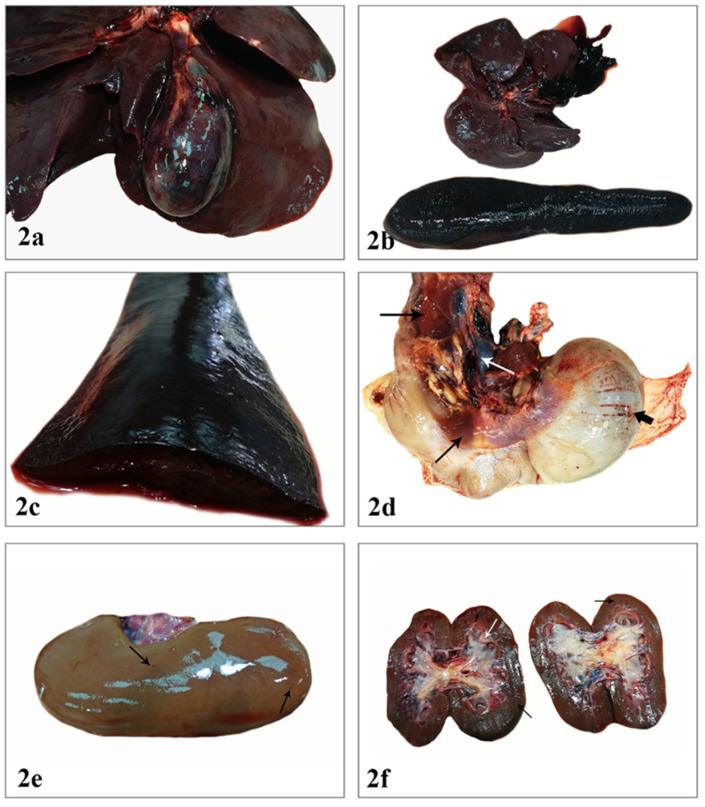
(**a**). The gallbladder is distended with multiple detectable subserosal hemorrhages. (**b**). Severe and homogenous enlargement of the spleen. The gallbladder contents appear dark red and viscous mixed with blood. (**c**). The spleen on the cut surface is solid and hyperemic with a dark cherry color. (**d**). Gastric lymph nodes are enlarged and dark red in color (white arrow). Note the linear subserosal hemorrhages (thick black arrow). Intense edema along the lesser curvature (thin black arrows). (**e**). Few scattered petechiae are observed on the surface of the renal cortex (arrows). (**f**). Sparse petechiae are present in the renal cortex, medulla, and the calyces of both kidneys (arrows).

During necropsy, external examination of the carcass revealed no changes except peripheral lymph node enlargement (Figure 1a). A few pinpoint hemorrhages involved the epiglottis while tonsils were slightly hyperemic (Figure 1b). Body condition, orifices, and skin were normal. Visceral lymph node enlargement mainly due to hyperplasia and/or hemorrhage was a constant finding. On the right side of the thoracic cavity, adhesions between the parietal and the visceral pleura were observed. Pulmonary edema was so severe that white frothy fluid was noticed up to the larynx region, while the caudal mediastinal lymph nodes were dark red in color and moderately swollen (Figure 1c). The pericardial cavity contained a small amount of serosanguineous fluid. Multiple petechial and ecchymotic hemorrhages were present on the left auricle and the subendocardial layer of the right ventricle (Figure 1d,e). In the abdominal cavity, abundant serosanguineous fluid with scattered fibrin fibers was observed (Figure 1f). Hemorrhages on the gallbladder wall and blood clots within the gallbladder lumen were observed (Figure 2a,b). The spleen was remarkably enlarged and hyperemic (Figure 2b,c). Furthermore, noticeable findings were the presence of linear serosal hemorrhages of the stomach as well as the intense gelatinous edema in the submucosa of its lesser curvature. Gastric lymph nodes were enlarged and dark red and black (Figure 2d). Moderately hyperplastic mesenteric lymph nodes were also noted. Finally, kidneys showed a few scattered petechiae throughout the cortex, in papillae and calyces (Figure 2e,f).

The lesions were compatible with those observed in septicemic and other diseases, such as erysipelas, salmonellosis, porcine reproductive and respiratory syndrome (PRRS), Aujeszky’s disease, pasteurellosis, ASF, classical swine fever (CSF), etc. Since, no pathognomonic macroscopic features are described to establish or rule out ASF, further laboratory investigation was requested to identify the causative agent. Immediately, the laboratory contacted the veterinary authorities of Central Macedonia and the Farm Animal Clinic (Swine Medicine and Reproduction Unit). Tissue samples were properly collected from lymph nodes, tonsils, lungs, heart, spleen, kidney, and liver and submitted to the National reference laboratory in Athens, Greece.

### 2.2. Clinical Presentation in the Farm

The backyard farm consisted of two linked subunits. The first housed unit included one boar, four sows, 13 piglets, and 11 fattening pigs. The second open-air unit was an olive grove where animals were introduced for grazing purposes. The grove was surrounded by an electrical fence. At the time of on-site confirmation, another 2 fattening pigs were found in subunit 2, thus a total of 31 animals were present on the whole farm. Based on the case history, fattening animals were moved from the housed to the open-air unit approximately one month prior to the dispatch of the sick animal to the laboratory. Three animals died with vague symptoms (diarrhea, anorexia) during the last 18 days prior to the ASF case recognition. The last dead animal was transferred to the Laboratory of Pathology of the Aristotle University of Thessaloniki for investigation [35].

According to information provided by the farmer, he was the only person with access to both subunits, whilst animals were fed corn from local producers and grazed in the olive grove (subunit 2). Moreover, the farmer reported that animals were not purchased from external sources during the past two years and a second empty backyard farm was identified at a close distance. However, according to an on-site inspection by local veterinary authorities, kitchen/food leftovers were identified in the olive grove, whilst the possible approach of subunit 2 by wild boars could not be excluded at least for the time period prior to the placement of the electrical fence. Also, the hypothesis of contact between animals and foreign personnel from a nearby greenhouse, which could be the source of food wastage provision to animals in subunit 2, could not be excluded also [35].

### 2.3. Laboratory Investigation

Laboratory testing was performed by the National Reference Laboratory (NRL) for African swine fever (Dept. of Molecular Diagnostics, FMD, Virological, Rickettsial and Exotic Diseases) located in Ag. Paraskevi, Athens, according to the official testing guidelines and EURL recommendations. The samples were received by the NRL on the 4th of February were immediately analyzed. Positive results were obtained on the evening of the same day, and the Chief Veterinary Officer was informed. On the 5th of February, the first positive case of ASF in Hellas was officially confirmed. In detail, testing for viral antigens was performed by a commercially available ELISA kit (Kit African Swine Fever Antigen; Ingenasa; Madrid; Spain). In addition, viral genome was detected by a commercially available real-time PCR assay (ID Gene™ African Swine Fever Duplex; IDvet; Grabels; France). ELISA testing for the ASF antigen demonstrated 7 positive samples out of 30 samples tested, and PCR-based testing indicated 12 out of 13 fattening pigs positive. In addition, anti-ASFV antibody detection was performed via a commercially available ELISA (ID Screen^®^ African Swine Fever Indirect; IDvet; Grabels; France), reporting 2 out of 31 samples positive and one sample as inconclusive [35,36] The methods used were according to those reported in the terrestrial manual of OIE [36].

### 2.4. Epidemiologic Results/Assessment

The same day, on February 5th, Veterinary and governmental authorities issued an alert about the ASF diagnosis. To avoid any potential contamination during the farmer’s entrance in the clinic premises disinfection was applied in the facilities of the Clinic and 7 pigs used for an experimental study in a stable at the Clinic were euthanized. Prior to euthanasia blood samples were collected, and results were negative for ASF. Simultaneously, a scientific committee was summoned by the authorities to manage the emerged issue. The committee visited the farm and under its supervision, clinical evaluation and blood samples were taken from the 31 animals of the farm for laboratory examination. Subsequently, immediate stamping out was performed, and all pigs were euthanized and appropriately buried at the site, whilst disinfection of the area was also performed. Additional control measures (creation of double surveillance zones, forbiddance of commercial use and transport of pigs and products thereof from and to the area of infection, etc.) according to the National contingency plan were taken into action in the respective prefecture in order to prevent the disease from spreading to neighboring disease-free farms and mainly in wild boar populations.

## 3. Discussion

In this outbreak, clinical signs did not strictly correspond to an expected acute ASF case [14,17]. Clinical signs could be attributed to several pathogens, thus differential diagnosis included a variety of possibilities. Based on the vague clinical picture—the gradual increase in mortality, and the proximity to a bordering region with active ASF cases—the possibility of an ASF case was among the probable causes in the differential diagnosis. Necropsy further revealed evidence that suggested ASF was a very possible etiology.

Pathological findings, although nonspecific, appeared to share some similarities with those described in an acute and subacute form such as hyperemic splenomegaly and nodal hematomas [14,15,17]. Nevertheless, pulmonary edema has been previously demonstrated in the acute form [14], and it has also been described along with ascites in the subacute form of the disease [17]. A significant observation was the absence of skin erythema or cyanosis, the sparsity of renal petechiae, and the detection of hematoma-like lymphadenitis only in the gastric and caudal mediastinal lymph nodes. This observation, in combination with the animals testing positive for ASF, but not displaying clinical signs of the lesions described in chronic disease, led us to suggest that the current disease might correspond to a less virulent strain [31,37]. In the absence of findings related to the chronic form of the disease and secondary infections, adhesions detected in the right thoracic cavity were probably not attributed to ASF. A previous trauma incidence was a more probable explanation.

There are a few swine diseases with high mortality, similar clinical signs, and gross lesions as in ASF, but the most remarkable similarities are observed in CSF from which ASF cannot be differentiated by clinical or postmortem examination. However, the ASF virus is unrelated to the classical swine fever virus (CSFV), and there is no cross-protection conferred by infections from CSFV and ASFV. Further differential diagnoses include erysipelas, salmonellosis, pasteurellosis, and other septicemic diseases [8].

## 4. Conclusions

Our report provides evidence of ASF cases with vague clinical symptoms and necropsy findings that could be attributed to ASF and other septicemic conditions. Based on the findings, in this case, it is strongly advised to carefully judge the clinical picture even in cases with mild symptoms, especially in areas neighboring high ASF-risk areas. The contamination source could not be fully evidenced in this case; thus, an alert is needed in cases of possible contact with wild boars in the backyard or housed units. Appropriate application of respective farm biosecurity measures should be the major priority in order to reduce the risk of infection. Finally, continuing efforts to develop an effective vaccine have shown promising results and should probably further contribute to the control of the disease [13].

## Data Availability

African Swine Fever Current situation in Greece. Meeting of Standing Committee on Plants, Animals, Food and Feed-Section: “Animal health and animal welfare”. Brussels, Belgium 13–14 February 2020. https://ec.europa.eu/food/system/files/2020-02/reg-com_ahw_20200213_asf_grc.pdf accessed on 21 February 2021.
